# Genetic signatures of natural selection in a model invasive ascidian

**DOI:** 10.1038/srep44080

**Published:** 2017-03-07

**Authors:** Yaping Lin, Yiyong Chen, Changho Yi, Jonathan J. Fong, Won Kim, Marc Rius, Aibin Zhan

**Affiliations:** 1 Research Center for Eco-Environmental Sciences, Chinese Academy of Sciences, 18 Shuangqing Road, Haidian District, Beijing 100085, China; 2 University of Chinese Academy of Sciences, 19A Yuquan Road, Shijingshan District, Beijing 100049, China; 3 Marine Biodiversity Assessment and Management Team, National Marine Biodiversity Institute of Korea, 101-75 Jangsan-ro, Janghang-eup, Seocheon-gun Chungcheongnam-do 33662, Korea; 4 Science Unit, Lingnan University, 8 Castle Peak Road, Tuen Mun, New Territories, Hong Kong, China; 5 School of Biological Sciences, College of Natural Sciences, Seoul National University, Seoul 08826, Korea; 6 Ocean and Earth Science, National Oceanography Centre, University of Southampton, European Way, Southampton SO14 3ZH, United Kingdom; 7 Department of Zoology, University of Johannesburg, Auckland Park, 2006, Johannesburg, South Africa

## Abstract

Invasive species represent promising models to study species’ responses to rapidly changing environments. Although local adaptation frequently occurs during contemporary range expansion, the associated genetic signatures at both population and genomic levels remain largely unknown. Here, we use genome-wide gene-associated microsatellites to investigate genetic signatures of natural selection in a model invasive ascidian, *Ciona robusta*. Population genetic analyses of 150 individuals sampled in Korea, New Zealand, South Africa and Spain showed significant genetic differentiation among populations. Based on outlier tests, we found high incidence of signatures of directional selection at 19 loci. Hitchhiking mapping analyses identified 12 directional selective sweep regions, and all selective sweep windows on chromosomes were narrow (~8.9 kb). Further analyses indentified 132 candidate genes under selection. When we compared our genetic data and six crucial environmental variables, 16 putatively selected loci showed significant correlation with these environmental variables. This suggests that the local environmental conditions have left significant signatures of selection at both population and genomic levels. Finally, we identified “plastic” genomic regions and genes that are promising regions to investigate evolutionary responses to rapid environmental change in *C. robusta*.

Micro-evolutionary processes, such as rapid local adaptation, represent key functional responses to environmental change^1^. The study of causes and consequences of local adaptation in response to changing environments can reveal mechanisms underlying population fitness in the wild^2^, which is fundamental to ecological and evolutionary studies. As invasive species can rapidly colonize a variety of dramatically different environments^3^, they represent promising models for studying selection that may promote rapid local adaptation associated with rapid environmental change. Despite growing research efforts to understand the role of selection in determining rapid microevolutionary processes over contemporary timescales^2^, it remains largely unknown how local environments mold genomes and population genetic structure of invasive species that successfully colonize a wide range of habitats and environmental conditions.

Although local adaptation is primarily triggered by selective pressures associated with non-parental environments^1^, understanding its causes and consequences during contemporary range expansions can be challenging^4^. Specifically, distinguishing the effects of selection from population history is often difficult due to the interplay between selection and population-associated processes (e.g. genetic drift and gene flow). Population-associated processes may mimic, weaken, or completely eliminate signatures of natural selection^5^,^6^. In addition, it is important to consider environmental factors that may cause new mutations and/or standing genetic variation^7^,^8^, and ultimately lead to changes in selection pressures and/or regimes (e.g. hard and soft selective sweeps). Consequently, the influence of environmental factors on local adaptation can be complex and variable in time and space, particularly in habitats such as aquatic ecosystems that often face rapidly changing conditions^3^. As a result, studies aiming at understanding the causes and consequences of local adaptation should consider different environmental niches and selection pressures and select a sound model system to comprehensibly understand the genetic mechanisms involved in local adaptation.

The changes of environmental conditions during biological invasions can be orders of magnitude higher and/or faster than what species would experience due to natural processes (e.g. seasonal fluctuations) in their native habitats^9^. Compared with terrestrial species, marine invasive species may experience stronger natural selection, due to their large population size and high levels of genetic diversity^10^. The highly invasive ascidian *Ciona robusta* (=*C. intestinalis* spA^11^) provides an excellent marine model species for the study of selective pressures contributing to rapid local adaptation under changing environmental conditions^12^. *C. robusta* encounters high selection pressures as it has rapidly spread to different environments and has survived a wide range of water temperature and salinity^9^. Recent taxonomic revision suggested *C. robusta* as native to Northwest Pacific. In the end of the 19^th^ century, *C. robusta* was first reported in the Mediterranean Sea, and since 1950 s, *C. robusta* expanded along the coasts of Australia, New Zealand, South Africa and South America^13^,^14^. When compared to other common invasive ascidians, *C. robusta* has wider thermal tolerance across multiple life-history stages^15^. Typically, *C. robusta* populations are large and contain an extremely high rate of nucleotide and structural polymorphisms^16^,^17^. In addition, *C. robusta* has a high per-year mutation rate (2–6 times higher than vertebrates^16^). These biological and genetic attributes can potentially contribute to directional selection on standing genetic variation and/or new mutations by both providing a source of beneficial alleles and avoiding the impact of genetic drift^7^,^8^,^10^. Another aspect that makes *C. robusta* an attractive model is that it has a small genome (160 MB) that has already been sequenced and assembled^17^,^18^. Taken together, *C. robusta* is a promising model to study genetic mechanisms of natural selection associated with rapid environmental change^12^.

Here, we study genetic signatures of *C. robusta* at population and genomic levels across a range of environmental conditions. Microsatellites within genes (i.e. gene-associated microsatellites) are often subjected to stronger selection than other regions because of their significant functions in regulating gene expression. We surveyed genome-wide gene-associated microsatellites derived from expressed sequence tags (ESTs), and selected representative loci that are evenly distributed across the genome to cover a wide range of functional genes^19^. By analyzing global populations that face dissimilar environmental conditions, we compared genome-wide patterns of population genetic differentiation and identified genomic regions that may be under selection during contemporary range expansions. Subsequently, we identified “plastic” genomic regions and genes to provide a framework to test what genes are involved in adaptation under two crucial environmental factors in the marine realm, temperature and salinity.

## Results

### Representative populations

Based on the obtained environmental gradient (Appendices S1-S4, Supporting information) and global population genetic analyses from previous studies^13^,^20^, we selected five representative populations along coastlines of four continents - two from Europe (Arenys de Mar, Spain [AM] and Blanes, Spain [BL]; high salinity and temperature) and the other three from Asia (Gampo, Korea [GAP]; low salinity and high temperature), Africa (Cape Town, South Africa [SA]; low salinity environment with low seawater temperature fluctuation) and Australasia (Nelson, New Zealand [NMF]; low salinity and temperature; Table 1; Appendix S2, Supporting information). *C. robusta* populations were randomly collected from the surface of artificial substrates at each site and preserved into absolute ethanol immediately after collection. We randomly chose an average of 30 individuals per population for genetic analyses.

### Population genetic structure

Out of 4,654 genome-wide gene associated microsatellites derived from 684,393 expressed sequence tags (ESTs), 218 polymorphic loci were selected (see more details in Lin *et al*.^19^), of which 177 loci (81.2%) were relatively evenly distributed across the 13 chromosomes and 41 loci (18.8%) were on scaffolds based on the KH assembly by Satou *et al*.^18^. Among them, 192 (88.1%) were successfully annotated (see more details in Lin *et al*.^19^) and 76 (34.9%) had Gene Ontology (GO) annotations which covered a wide range of functional genes based on GO terms (Appendix S5, Supporting information). By checking pooled samples as recommended by Thomas *et al*.^21^, we discarded loci that were not likely to be under selection.

A total of 152 candidate microsatellites were re-genotyped individually for all populations. The number of alleles (*A*) ranged from two to 20, allelic richness (*A*_R_) varied from 1.337 to 10.035, observed heterozygosity (*H*_O_) ranged from 0 to 0.798, and expected heterozygosity (*H*_E_) varied from 0.027 to 0.872 (Appendix S6, Supporting information). A total of 43, 20, 33, 18 and 31 loci significantly deviated from Hardy-Weinberg equilibrium in the populations AM, BL, SA, NMF and GAP, respectively. Since null alleles were detected at two loci (Cin170 and Cin171) across all populations, we excluded these two loci for subsequent analysis.

**Figure 1:**
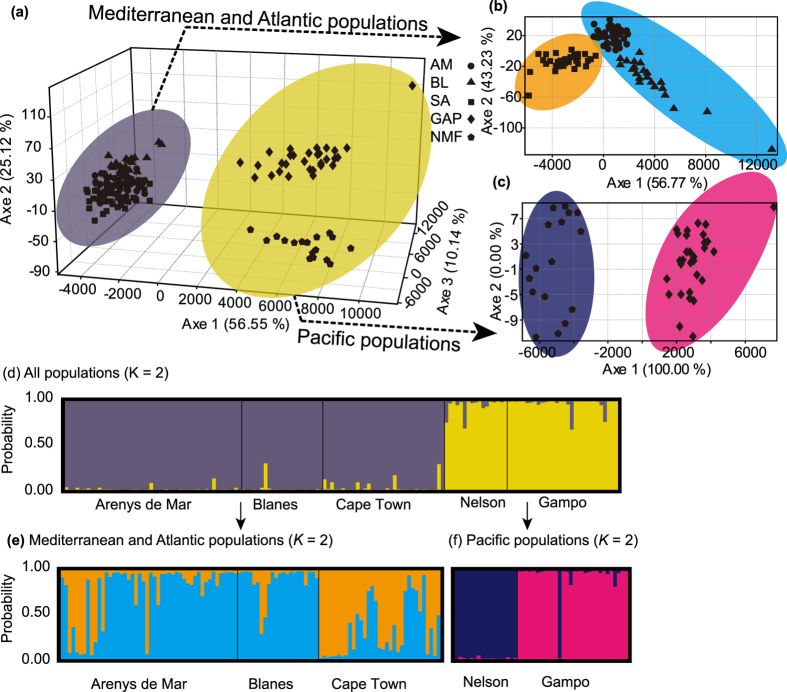
Three-dimensional factorial correspondence analysis (3D-FCA) and individual Bayesian assignment proportions determined using STRUCTURE for all populations (**a**,**d**), Mediterranean Sea and Atlantic Ocean populations (**b**,**e**) and Pacific Ocean populations (**c**,**f**) based on all neutral polymorphic microsatellites. For Bayesian clustering analysis (**d**,**e** and **f**), each genotype is represented by a thin vertical line, with proportional membership in different clusters. Bold vertical lines separate collection sites.

Our microsatellite data showed a relatively high level of genetic differentiation among populations. The lowest levels of genetic differentiation (*F*_ST_ = 0.0169) were detected between the two European populations (AM & BL), while the highest (*F*_ST_ = 0.2041) were detected between the population pair from Europe (AM) and Australasia (NMF; Table 2). Populations from the Pacific Ocean (NMF and GAP) were highly differentiated from those collected from the Mediterranean Sea and Atlantic Ocean (AM, BL and SA; *F*_ST_ = 0.1834 − 0.2041). Similarly, the 3D-FCA divided the populations into two groups - group 1 from the Pacific coast (NMF and GAP) and group 2 from the Mediterranean Sea and Atlantic coast (AM, BL and SA; Fig. 1a). When the Mediterranean-Atlantic population group and Pacific population group were analyzed separately, populations within each group were well separated based on their geographical origins. For the Mediterranean-Atlantic population group, two Mediterranean populations were grouped together, while the other populations clustered with the one from South Africa (Fig. 1b). For the Pacific population group, populations from Korea and New Zealand were divided into two clusters (Fig. 1c). Bayesian probability assignment conducted in STRUCTURE revealed two genetically divergent clusters (Fig. 1d), corresponding to their clustering assignment based on 3D-FCA (Fig. 1d). When the Mediterranean-Atlantic and Pacific groups were considered separately, we detected similar results to those found using 3D-FCA (Fig. 1e and f). In order to consider the potential influence on the genetic structure of selected populations based on loci that might be under selection, we analyzed the population genetic structure using only putatively neutral loci, and obtained similar results (see Appendix S7, Supporting information).

### Loci under selection

**Figure 2:**
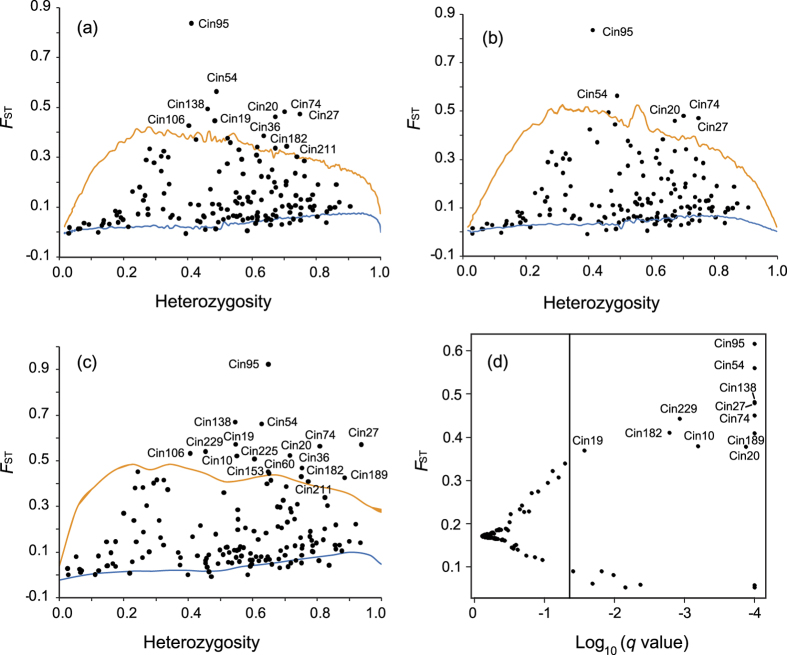
Outlier detection at 150 microsatellite loci among five populations with programs LOSITAN under infinite alleles model (**a**), LOSITAN under stepwise mutation model (**b**), ARLEQUIN (**c**) and BAYESCAN (**d**). Solid vertical line in (d) represents false discovery rate of 0.05. Orange and blue lines in (**a**), (**b**) and (**c**) represent upper and lower 95% confidence intervals, respectively.

To identify loci under selection among all populations and between population pairs, we adopted three theoretical approaches (LOSITAN, ARLEQUIN and BAYESCAN, see details in Methods section) based on the *F*_ST_-outlier test for modeling neutral loci. When tests were performed based on all populations, a total of 41 loci were identified under selection: 19 (8.7%) candidates for directional selection (Table 3; Appendices S8–S9, Supporting information) and 22 (10.1%) candidates for balancing selection (Appendices S10–S11, Supporting information). Under the IAM model, the LOSITAN analysis detected signatures of directional selection at 11 loci (5.0%) and balancing selection at 13 loci (6.0%) among all five populations (Table 3; Fig. 2). Under the SMM model, five (2.3%) and 19 (8.7%) loci were considered to be under directional selection and balancing selection, respectively (Table 3; Appendix S10, Supporting information). Of the 19 candidates for directional selection, 13 were detected as outliers based on BAYESCAN and 17 based on ARLEQUIN. Eight loci were consistently identified as outliers with all three methods (95% confidence). Three loci (Cin60, Cin153 and Cin225) were identified as putatively under directional selection only with the hierarchical island model.

When we conducted the pairwise population analysis using the BAYESCAN method, a total of 10 and six loci were indicated to be under directional selection and balancing selection respectively, four of which (Cin8, Cin124, Cin97 and Cin204) were not detected when all populations were considered. In particular, seven loci (Cin20, Cin27, Cin54, Cin74, Cin95, Cin138 and Cin189) were potentially under directional selection in different population pairs (Appendix S9, Supporting information). Interestingly, all outlier loci were associated with the population from New Zealand and/or the one from Korea (Appendix S8, Supporting information).

**Figure 3:**
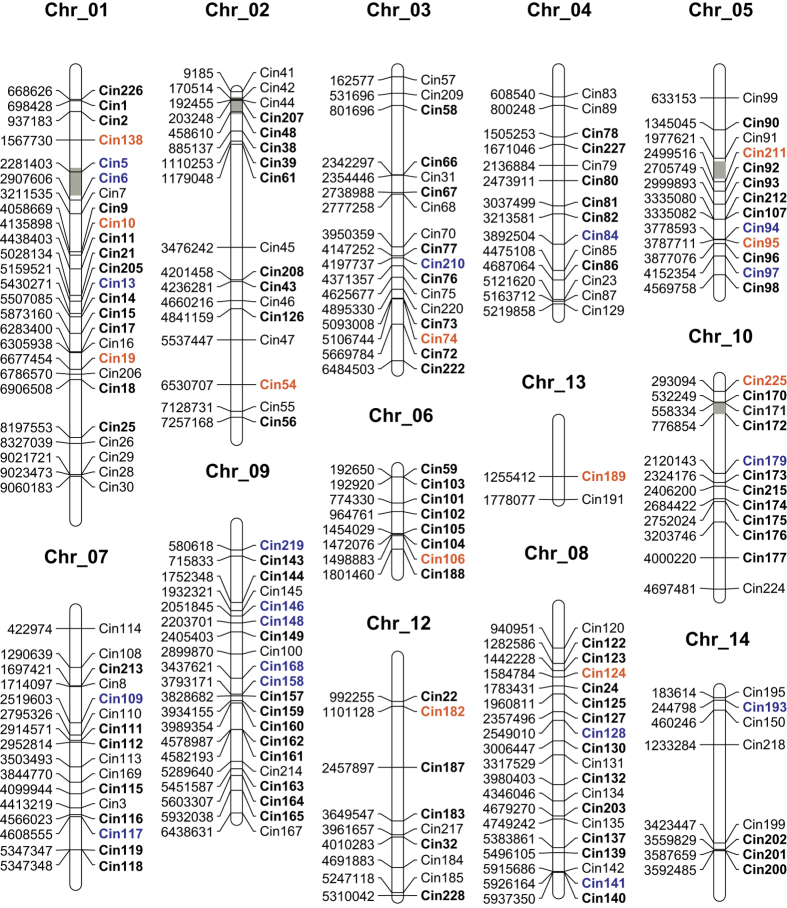
The chromosomal locations of the 177 gene-associated microsatellite markers used in this study. Numbers on the left represent the chromosomal location for each microsatellite marker based on the KH assembly of Satou *et al*.^18^. The names of microsatellite markers are labelled on the right. Bold names indicate loci used for genome scan for all individual in all populations; candidate loci for directional selection (orange) and balancing selection (blue) are highlighted. Regions revealed as hotspots of introgression by Roux *et al*.^39^ are indicated by gray boxes.

Of the 41 candidate loci under selection, 30 loci (12 directional and 18 balancing) were located on nine different chromosomes (Fig. 3), while the remaining 11 loci (seven directional and four balancing) were located on ten different scaffolds. Interestingly, among the 12 candidates of directional selection loci on chromosomes, all neighboring markers were not affected. For example, the locus Cin73 was detected under directional selection, but the nearest locus Cin74 (13.7 kb) was detected as neutral (Fig. 3). Similarly, the selection-neutral pattern was detected among several loci pairs such as Cin104-Cin106 (26.8 kb; Fig. 3). When we surveyed the length of selective sweep regions, some regions were shorter than 8.9 kb (Fig. 3).

For the 18 loci considered to be under balancing selection, five were located on the chromosome 9, while the remaining 13 loci were scattered on eight chromosomes. Similarly, the length of balancing selection on chromosomes was relatively short; for example, Cin141 and Cin158 were identified as balancing selection loci, but their nearest loci (Cin140, 11.2 kb and Cin168, 35.5 kb) were identified as neutral (Fig. 3). Although the balancing selection is considered as an important selective force in evolution, there are still methodological and technical issues in detecting balancing selection in hitchhiking mapping, especially hindered by the high rate of false positives^22^,^23^. Consequently, the below analyses focus on only footprints of directional selection.

### Genes in selective sweep windows

**Figure 4:**
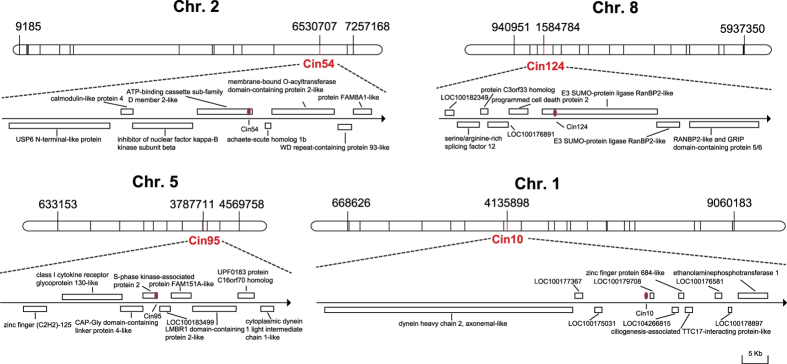
Four examples of the genes under directional selection in the selective sweep windows within a 20-kb distance up- and down-stream. The location for each gene was assessed by BLAST against the KH assembly of Satou *et al*.^18^.

We found a total of 132 genes that were located in all selective sweep windows (Appendix S12, Supporting information). Based on known functions, many of these genes are crucial in adaptation to harsh environments and/or environmental changes, such as the calmodulin-like protein 4 (CALML4) in the Cin54 selective sweep window, the ciliogenesis-associated TTC17-interacting protein-like and dynein heavy chain 2 (CATIP2) in the Cin10 selective sweep window, and the programmed cell death protein 2 (PDCD2) in the Cin124 window (Fig. 4).

### Correlation between environmental factors and genetic variation

When the SAM analysis was performed to identify possible correlation between microsatellite alleles and environmental variables, 16 putatively selected loci were significantly correlated with at least one environmental variable, and among these, 14 were under directional selection in the global outlier analysis (Table 3). The remaining two loci were likely under directional selection and balancing selection in the pairwise analysis, respectively (Appendix S13, Supporting information). Amongst the neutral loci, 17 were identified to be associated with at least one environmental variable (Appendix S13, Supporting information).

## Discussion

In this study, we used genome-wide gene-associated microsatellites to reveal genetic signatures of natural selection at both population and genomic levels in the model invasive ascidian *C. robusta*. We found significant genetic differentiation among populations collected from different geographical regions and recovered multiple genomic regions likely to be under selection. We then analyzed six key environmental factors in relation of these genomic regions and found candidate genes that were associated with temperature and salinity changes in different habitats. Thus, these candidate genes might have an important role in rapid local adaptation during range expansions. Taken together, our results suggest that rapid local adaptation is a major driver for significant population differentiation, and that “plastic” genomic regions represent promising regions for future studies aiming at better understanding rapid adaptation in invasive species.

We found significant genetic structure among populations, with a wide range of *F*_ST_ values (0.0169–0.2041; Table 2; Fig. 1). These results were similar to a previous study that detected a high level of genetic differentiation among populations at a regional scale (Pacific coast of North America)^20^. Given a possible high level of gene flow on the Pacific coast of North America mediated by frequent shipping, our results suggest that rapid local adaptation played a key role in determining the observed patterns (e.g. rapid local adaptation hypothesis^20^). Although we could not rule out other possibilities such as different introduction sources and/or demographic processes, available evidence, such as differentiation high level of genetic homogeneity across the major distribution ranges at the global scale^14^,^20^ and no genetic bottleneck detected in multiple studies^13^,^20^ (see also Table 1), suggests that the above factors should not be the major drivers for the detected patterns across multiple studies.

The high population genetic differentiation found among certain populations is likely driven by selection as a result of local environmental conditions that may have selected pre-adapted genotypes, facilitating rapid genetic divergence among populations that have high levels of gene flow^20^. Additionally, we found strong signals of natural selection. Out of 218 microsatellite markers, 19 were considered to be under directional selection (Table. 3). All these putatively selected loci provided finer population structure than the neutral markers. For example, Bayesian clustering analysis (Appendix S7, Supporting information) showed that individuals GAP8-2 and GAP17-2 were admixed between two clusters according to neutral loci, whereas these two individuals were undoubtedly assigned to the Pacific cluster when we considered loci likely under selection. Remarkably, our results suggest that a few loci influenced by selection can highlight the existence of important population structure^24^ and provide more details of genetic patterns^25^. We further identified loci inferred to be under directional selection that were associated with salinity and water temperature (Table 3), suggesting that environmental factors such as salinity and water temperature can directly influence the detected genetic divergence.

Studies have shown that environment-driven selection can leave significant signatures in species in a few generations. For example, 20 generations were enough for an invasive species to show genetic divergence^26^, and less than 13 generations were needed to trigger crucial processes during ecological speciation such as reproductive isolation^27^. For *C. robusta*, selection can occur in a short period of time, mainly owing to its biological characteristics such as the rapid growth rate, relatively high fecundity and short life cycle. *C. robusta* can have two or three generations each year, and new generations can reach sexual maturity in approximately two months^28^,^29^. *C. robusta* can produce gametes continually as long as water temperature is suitable, and each mature individual can potentially spawn daily approximately 500 eggs^12^. In addition, a high mutation rate and large effective population size provides a high level of genetic variance for divergent selection^30^ to occur. Rapid selection over a short time scale can leave significant signatures in *C. robusta* populations with a recent invasion history such as those in New Zealand (1950s^14^) and South Africa (mid 20^th^ century^14^).

Interestingly, we found the effect of a selective sweep on the genomic regions of *C. robusta* less than 8.9 kb (Fig. 3). Similar studies have identified that selective sweeps in genomic regions ranged from less than 10 kb to hundreds of kilobases^31^. The length of sweep regions can be affected by several factors, including the intensity of selection pressure, type of selection (hard versus soft sweeps), and several genomic features such as recombination rate^8^.

Identifying selection pressure and its intensity behind the observed selective signatures is still a major conundrum. So far, only a handful of environmental factors driving selection have been identified, such as climate warming/cooling^32^. In marine ecosystems, environmental factors such as water temperature, salinity and pH are known to be critical for organisms^4^. However, it remains extremely challenging to assess both the relative role of a single factor and combined effects of multiple factors, as well as possible effects of selection intensity on the length of selective sweep regions on chromosomes^3^. Our study showed that 15 loci (Table 3; Appendix S13) were subjected to directional selection associated with salinity and temperature changes indicating the possibility that salinity and temperature adaptation may facilitate marine invasions. Despite recent progress in this research area, long-term investigations based on both wild populations and common garden experiments are largely needed to clarify possible mechanisms on how organisms genetically respond to rapidly changing environments and how different selection pressures determine the length of selective sweeps on chromosomes.

Regarding types of detected selection, two evolutionary hypotheses have been proposed to explain how populations adapt to novel environments during biological invasions: selection on new mutations and selection on standing genetic variation^8^. For the former model, a novel and major effect mutation arises on a single haplotype in a population and ultimately reaches fixation (hard selective sweep), while for the latter model, selection acts on beneficial alleles present on many haplotypes in a population with standing genetic variation (soft selective sweep). Usually, soft selective sweeps need a high level of genetic diversity to generate higher frequency of beneficial alleles for selection. To date, mounting evidence indicates that hard selective sweeps might not be the dominant mode of adaptation in many species^33^,^34^. Based on results from computer simulations, soft sweeps were expected to strongly affect genomic regions less than 10 kb when selection coefficients were ≥0.05^35^. However, it should be noted that the length of selective sweep regions left on genomic regions by both types of selections highly depends on the recombination rate^36^. The high recombination rate can largely narrow down the hitchhiking effect, and available examples showed that the genome length could be limited less than 1 kb in genomic regions with a high recombination rate^36^.

In general, *C. robusta* has high levels of genetic diversity in populations, large effective population sizes and a relatively strict outcrossing breeding model to avoid diversity decrease in natural populations^16^,^17^,^20^. All these biological characteristics can facilitate a gradual rise in the frequency of favorable alleles when soft selective sweeps occur during biological invasions. In addition, the high recombination rate across the whole genome^37^ can narrow down the affected genomic regions by soft selective sweeps, leading to the patterns observed in our study. However, our analyses here could not exclude the possibility of hard selective sweeps. *C. robusta* has an extremely high per-year mutation rate^16^, which may provide enough mutations for hard selective sweeps.

In our study, we detected 19 out of 218 microsatellite loci (8.7%) under directional selection (Table 3; Fig. 2). Eight loci (3.7%) were indicated to be under directional selection with all three methods employed, and the percentage is similar to previous studies that have detected an average of 5% (range: 2.8–15.0%^38^). Given that recent work has identified and mapped ‘hot spots of introgression’ in the *C. robusta* genome^39^, intrinsic genetic incompatibilities may provide an alternative explanation for generating and maintaining observed selected loci^40^, although the outlier loci detected in our study avoid the hotspots of introgression (Fig. 3). In addition, various other processes may lead to *F*_ST_ outliers, such as loci from regions with different recombination rates and demographic patterns (see review by Bierne *et al*.^40^). In addition, the programs used to detect outlier loci were sensitive to admixed individuals and demographic variation of populations (e.g. range expansion), consequently deep investigation is need at the putatively selected loci to confirm their adaptive contribution by further experiments.

When we analyzed allele-association with temperature and salinity, SAM analyses identified 16 putatively selected loci - 15 were considered to be under directional selection and one was indicated to be under balancing selection. When the sequences of these16 loci were subjected for BLAST, 13 were successfully annotated, some of which have crucial functions, such as carboxypeptidase Z-like (CPZ) regulates crucial aspects of development^41^, and polyadenylate-binding protein 2-like (PABP2) is an important regulator of mRNA translation and stability^42^. These results suggest that these genes, and/or surrounding ones on chromosomes, may be involved in responding to rapid salinity and/or temperature changes.

In addition, 15 putatively directional selection loci were located within genes, and many of these genes are crucial for physiological processes (Table 3; Appendix S12, Supporting information). For example, the locus Cin95 was found within the S-phase kinase-associated protein 2 (SKP2) gene (Fig. 4). Interestingly, S-phase kinase-associated proteins were responsible for sperm activation in *Ciona*^43^, and related studies found that the degree of sperm mobility was considered the adaptive response to salinity in the Baltic cod, *Gadus morhua*^44^. Moreover, we found that 117 genes were tightly linked to the outlier microsatellite loci (Appendix S12, Supporting information). Although the putative functions of some genes were unknown, the ones with known functions were associated with the change of environmental factors. For example, the CALML4 gene closely linked with Cin54 (Fig. 4) was involved in the interaction with calcium ions (Ca^2+^) and the regulation of diverse signaling pathways^45^. All the results suggest that these genes, either by themselves or through gene networks, play roles in rapid local adaptation, and further investigations are needed to clarify their roles in functions, regulations and pathways.

The *F*_ST_ values obtained here were comparative to our previous global genetic study, where population structure was assessed using 12 randomly selected genomic microsatellites based on the same populations^20^. However, all analyses in our previous study did not recover significant population structure^20^. In contrast, significant population genetic structure was detected consistently across multiple analyses in this study (Table 2; Fig. 1). Such difference is mainly due to the different scales of genetic markers used (genome-wide versus 12 randomly selected microsatellites) and different types of microsatellites used (gene-associated versus genomic microsatellites).

Usually, a large number of genome-wide set of markers has a higher power in resolving population structure^46^, and the use of gene-associated markers could better identify patterns of genetic differentiation^25^,^47^,^48^. Compared to genetic markers located in “junk regions”, the gene-associated markers are more prone to divergent selection, leading to the increase of genetic differentiation among populations but decrease of variability within populations, while the markers in “junk regions” experience much less frequent divergent selection, resulting in a low power to assess weak but significant population genetic structure^48^. Consequently, gene-associated markers and markers in “junk regions” often reveal contrasting patterns of population genetic differentiation^25^. The highest *F*_ST_ between AM and NMF was 0.2041 (Table 2), suggesting that historical selection events may have left residual signals on the neutral gene-associated markers^25^. The effect of selection can persist for approximately 2,900 generations with effective population size assumed infinite and a selection coefficient of 1% in *Drosophila*^5^,^33^. Therefore, the gene-associated markers that we used provided high resolution to resolve population genetic structure, especially considering the high levels of gene flow among populations^24^ that exist among populations of the studied species.

## Methods

### Selection of representative populations

To select representative populations living in different environments, we analyzed key environmental variables (i.e. water temperature and salinity)^15^ across the reported distribution range of *C. robusta*. We obtained annual average and monthly temperature and salinity data from World Ocean Atlas 2013 of the National Oceanic and Atmospheric Administration (NOAA; http://www.nodc.noaa.gov/OC5/SELECT/woaselect/woaselect.html; Table 1; Appendices S1 and S2, Supporting information) for the period of 1955 to 2012. We used a nonparametric test (Mann–Whitney *U* test) in SPSS v.18 to test differences in temperature and salinity among sampled sites (Appendix S3, Supporting information). We also performed a principal component analysis (PCA) to illustrate environmental differences among populations (Appendix S4, Supporting information).

### Selection of gene-associated microsatellites and microsatellite genotyping

Genomic DNA was extracted from approximately 10 mg of siphon tissue using the proteinase K method as described by Waters *et al*.^49^. The polymorphic loci based on Lin *et al*.^19^ were first evaluated in the selected five populations using the DNA pooling strategy following Thomas *et al*.^21^. Populations were represented by five unique pool samples where DNA samples were normalized to 15 ng/μL from all individuals in a given population. Subsequently, we genotyped 218 microsatellite loci using the five population pools and an individual was randomly selected for comparison as each locus showed characteristic slippage patterns^21^. The fragment patterns were visually inspected by pairwise comparison between populations as recommended by Thomas *et al*.^21^ to identify the candidate loci under selection. These candidate loci were subsequently re-genotyped individually for each population to confirm their selective signatures. Polymerase chain reactions (PCRs) were performed in a three-primer system following the protocol of Schuelke^50^. All forward primers were 5′-tailed with the M13(−29) forward sequence (5′-CACGACGTTGTAAAACGAC-3′) and used in combination with an M13 primer of the same sequence but 5′-labelled with 6-carboxyfluorescein (6-FAM), hexachlorofluorescein (HEX), carboxytetramethylrhodamine (TMR) or carboxy-X-rhodamine (ROX). PCRs were carried out in 96-well plates with 12.5 μL reaction volume containing approximately 40 ng of genomic DNA, 1 × PCR buffer, 0.2 mM of each dNTP, 1.5 mM of Mg^2+^, 0.5 pmol M13-tailed forward primer, 1 pmol reverse primer and 1 pmol fluorescently labeled M13 primer, and 0.25 unit of *Taq* DNA polymerase (Takara Bio Inc.) with the same cycling profiles as in Lin *et al*.^19^ Amplification products were separated by ABI 3730xl automated sequencer (Applied Biosystems, Foster City, CA, USA) with GeneScan^TM^-500 LIZ^TM^ size standard (Applied Biosystems). Genotypes were scored using GeneMapper^TM^ software v. 4.0 (Applied Biosystems).

### Analyses of genetic variation

We calculated population genetic parameters including the number of alleles (*A*), observed heterozygosity (*H*_O_), expected heterozygosity (*H*_E_), and allelic richness (*A*_R_) using FSTAT v. 2.9.3.2^51^. The presence of null alleles was assessed using MICRO-CHECKER v. 2.2.0^52^. The probability of significant deviation from Hardy-Weinberg equilibrium (HWE) and fixation index (*F*_IS_) were evaluated using the Markov chain-based method (10,000 dememorization steps, 500 batches and 5,000 iterations per batch^53^) implemented in GENEPOP v. 3.4^54^. We computed the *q*-values to adjust the *P*-values for multiple tests, using the QVALUE package in *R*^55^.

Population genetic differentiation was determined by *F*_ST_ (*θ* estimator) for all population pairs using GENEPOP v. 3.4^54^. Exact tests for population differentiation (10,000 dememorization steps, 500 batches and 5,000 iterations per batch) were performed for all population pairs. To further investigate patterns of population structure, we performed the Bayesian clustering analysis implemented in STRUCTURE v. 2.3.1^56^. The admixture model was applied with five replicate chains of 1,000,000 Markov Chain Monte Carlo (MCMC) iterations and 100,000 burn-in repetitions, with values of *K* = 1–5. The most likely number of clusters was determined by obtaining the maximum value of Δ*K* proposed by Evanno *et al*.^57^. We used DISTRUCT v.1.1 to visualize the individuals’ assignment to different clusters^58^. In addition, we performed the three-dimensional factorial correspondence analysis (3D-FCA) using GENETIX v. 4.05^59^.

### Detection of loci under selection

In this study, we employed the hitchhiking mapping strategy to identify loci under selection at the population level. Hitchhiking mapping is based on the principles of a selective sweep - when a gene is under selection, there can be a significant loss of diversity at not only target genes, but also linked genes because of the “hitchhiking” effect (i.e. selective sweep)^40^. Consequently, selective sweeps may skew the frequency distribution of polymorphism at specific genomic regions rather than overall loss of genetic diversity (as seen in population-level processes such as a genetic bottleneck). The genomic regions under selection can be identified by a multi-locus scan using genome-wide molecular markers such as microsatellites.

To identify loci under selection among all populations and between population pairs, we adopted three theoretical approaches based on the *F*_ST_-outlier test for modeling neutral loci. For the first method developed by Beaumont & Nichols^60^, we used the program LOSITAN to detect loci under selection. The method employs coalescent simulations to estimate the distributions of heterozygosity and *F*_ST_ under the island model^61^. Loci that do not fit neutral expectations are considered candidates of selection. A total of 100,000 coalescent simulations were carried out to generate *F*_ST_ values under both the infinite alleles model (IAM) and stepwise mutation model (SMM). As recommended by Antao *et al*.^61^, we used 95% and 99% confidence intervals and false discovery rate (FDR) of 0.05 for both ‘neutral mean *F*_ST_’ and ‘force mean *F*_ST_’ options. The second hierarchical method was modified by Excoffier *et al*.^23^ from the methods developed by Beaumont & Nichols^60^, which assumes a hierarchical Island Model and is able to take hierarchical population structure into account. We performed this analysis using ARLEQUIN v.3.5.2.1^62^ under the hierarchical island model. The hierarchical analysis was carried out by assuming a model of 10 groups, with each consisting of 100 demes with 50,000 simulated loci. The third and final method used is based on the Bayesian model by Beaumont & Balding^22^ and decomposes *F*_ST_ values into locus-specific components (α) and population-specific components (β^51^) using BAYESCAN v. 2.1^63^. This method is based on a reversible jump MCMC algorithm and calculates the posterior probability of each under selection locus by assuming two alternative models (selection-based model and neutral model). Following 10 pilot runs of 5,000 iterations with a 50,000 burn-in and a thinning interval of 20, we used the prior odds of 10 in favor of a neutral mode.

### Analyses of selective sweep windows

As selective sweep regions were narrow in *C. robusta* (see the Results section for more detail), we analyzed selective sweep windows for 20 kb up- and down-stream of each locus potentially under selection. As demonstrated by many related studies^31^,^36^, the survey of relative narrow selective sweep windows can largely avoid false negatives. The genes within each selective sweep window were identified and analyzed based on the KH assembly.

### Correlation between environmental factors and genetic variation

We used SAM v.2^64^ to identify possible correlation between microsatellite alleles and environmental variables. Univariate linear regression analysis with the least-squares method was used to determine the relationship between the allelic frequency at each candidate microsatellite loci and the environmental variable at a critical level of 0.001. The annual average temperature and salinity data obtained from World Ocean Atlas 2013 of the National Oceanic and Atmospheric Administration (NOAA; http://www.nodc.noaa.gov/OC5/SELECT/woaselect/woaselect.html; Table 1) was used in SAM. We used a set of six climate variables, including of annual water temperature, lowest monthly average water temperature, highest monthly average water temperature, annual salinity, lowest monthly average salinity and highest monthly average salinity. The significance of associations was determined by Wald test.

## Additional Information

**How to cite this article**: Lin, Y. *et al*. Genetic signatures of natural selection in a model invasive ascidian. *Sci. Rep.* **7**, 44080; doi: 10.1038/srep44080 (2017).

**Publisher's note:** Springer Nature remains neutral with regard to jurisdictional claims in published maps and institutional affiliations.

## Supplementary Material

Supplementary Information

## Figures and Tables

**Table 1:** Basic information about sampling sites and measures of genetic variation.

Site					*A*_R_	Genetic variation		
Code	Region/state and country	*N*	Salinity (ppt)	Temperature (°C)		*H*_O_	*H*_E_	*F*_IS_
AM	Arenys de Mar, Spain	48	37.7	18.0	3.16	0.302	0.420	0.283
BL	Blanes, Spain	22	38.0	17.4	3.13	0.299	0.446	0.336
SA	Cape Town, South Africa	33	35.2	16.0	3.33	0.319	0.438	0.276
NMF	Nelson, New Zealand	17	34.8	13.6	4.19	0.394	0.559	0.303
GAP	Gampo, Korea	30	33.7	17.7	4.04	0.388	0.530	0.271

Annual average values for both salinity and temperature are shown here. *N* = the number of individuals; *A*_R_ = allelic richness; *H*_O_ = observed heterozygosity; *H*_E_ = expected heterozygosity; *F*_IS_ = inbreeding coefficient.

**Table 2:** Pairwise population genetic differentiation (pairwise *F*_ST_ estimates) based on 152 microsatellites markers.

	AM	BL	SA	NMF
BL	0.0169**			
SA	0.0318**	0.0474**		
NMF	0.2041**	0.1966**	0.2019**	
GAP	0.1958**	0.1834**	0.1973**	0.0874**

Population abbreviations as per Table 1. **P < 0.01.

**Table 3:** Summary of loci under directional selection in LOSITAN, BAYESCAN and ARLEQUIN analyses using the global analysis based on all populations, and results for correlation with environmental variables in the SAM test (*P* < 0.001, Bonferroni correction for multiple comparisons).

Locus	Annotation	LOSITAN		ARLEQUIN	BAYESCAN	SAM
		IAM	SMM			Association with environmental variables
Cin10	no hit	—	—	*	**	Min. sal.
**Cin19**	**no hit**	*	—	**	*	Min. temp., Min. sal.
**Cin20**	**protein MB21D2**	**	**	*	**	Ann. temp., Min. temp., Min. sal.
**Cin27**	**LOC100176860**	**	**	**	**	Min. temp., Ann. sal., Min. sal., Max. sal.
Cin36	flocculation protein FLO11	*	—	*	—	Min. temp., Ann. sal., Min. sal., Max. sal.
**Cin54**	**ATP-binding cassette sub-family D member 2-like**	**	**	**	**	—
Cin60	LOC104265676	—	—	*	—	Min. sal.
**Cin74**	**polyadenylate-binding protein 2-like**	**	**	**	**	Min, temp., Ann. sal., Min. sal.
**Cin95**	**S-phase kinase-associated protein 2**	**	**	**	**	—
Cin106	LOC100180535	*	—	*	—	Min. temp., Min. sal.
**Cin138**	**LOC100178806**	**	—	**	**	Min. temp., Ann. sal., Min. sal., Max. sal.
Cin153	IST1 homolog	—	—	*	—	Ann. sal., Min. sal., Max. sal.
**Cin182**	**LOC100184112**	*	—	*	**	—
Cin189	GPI-anchor transamidase	—	—	*	**	Min. sal.
Cin211	no hit	*	—	*	—	Min. temp., Ann. sal., Min. sal., Max. sal.
Cin225	persulfide dioxygenase ETHE1, mitochondrial-like	—	—	*	—	Min. sal.
Cin229	carboxypeptidase Z-like	—	—	*	**	Min. temp., Min. sal.

IAM, infinite alleles model; SMM, stepwise mutation model; SAM, spatial analysis method; *P < 0.05; **P < 0.01; –, not significant; Ann. temp., Annual water temperature; Min. temp., Lowest monthly average water temperature; Max. temp., Highest monthly average water temperature; Ann. sal., Annual salinity; Min. sal., Lowest monthly average salinity; Max. sal., Highest monthly average salinity. The loci detected consistently across all the three methods (i.e. LOSITAN, BAYESCAN and ARLEQUIN) are bolded.
