# Supplementary material for: Genetic signatures of natural selection in a model invasive ascidian
Source: Sci Rep. 2017 Mar 7;7:44080. doi: 10.1038/srep44080 (PMC5339779; doi:10.1038/srep44080)
Supplement: Supplementary Information [file srep44080-s1.doc]

**Genetic signatures of natural selection in a model invasive ascidian**

Yaping Lin^1,2^, Yiyong Chen^1,2^, Changho Yi^3^, Jonathan J. Fong^4^, Won Kim^5^, Marc Rius^6,7^, Aibin Zhan^1,2*^

^1^ Research Center for Eco-Environmental Sciences, Chinese Academy of Sciences, 18 Shuangqing Road, Haidian District, Beijing 100085, China.

^2^ University of Chinese Academy of Sciences, 19A Yuquan Road, Shijingshan District, Beijing 100049, China.

^3^ Marine Biodiversity Assessment and Management Team, National Marine Biodiversity Institute of Korea, 101-75 Jangsan-ro, Janghang-eup, Seocheon-gun Chungcheongnam-do 33662, Korea.

^4^ Science Unit, Lingnan University, 8 Castle Peak Road, Tuen Mun, New Territories, Hong Kong, China.

^5^ School of Biological Sciences, College of Natural Sciences, Seoul National University, Seoul 08826, Korea.

^6^ Ocean and Earth Science, University of Southampton, Southampton SO14 3ZH, United Kingdom.

^7^ Department of Zoology, University of Johannesburg, Auckland Park, 2006, Johannesburg, South Africa.

*** Corresponding author:** Dr. Aibin Zhan; Research Center for Eco-Environmental Sciences, Chinese Academy of Sciences, 18 Shuangqing Road, Haidian District, Beijing 100085, China; Email: [zhanaibin@hotmail.com](mailto:zhanaibin@hotmail.com) or [azhan@rcees.ac.cn](mailto:azhan@rcees.ac.cn); Phone & Fax: (+86)-10-6284-9882.

**Supporting information**

**Appendix S1** Monthly (a) temperature and (b) salinity data in sampling locations. Population names as per Table 1.


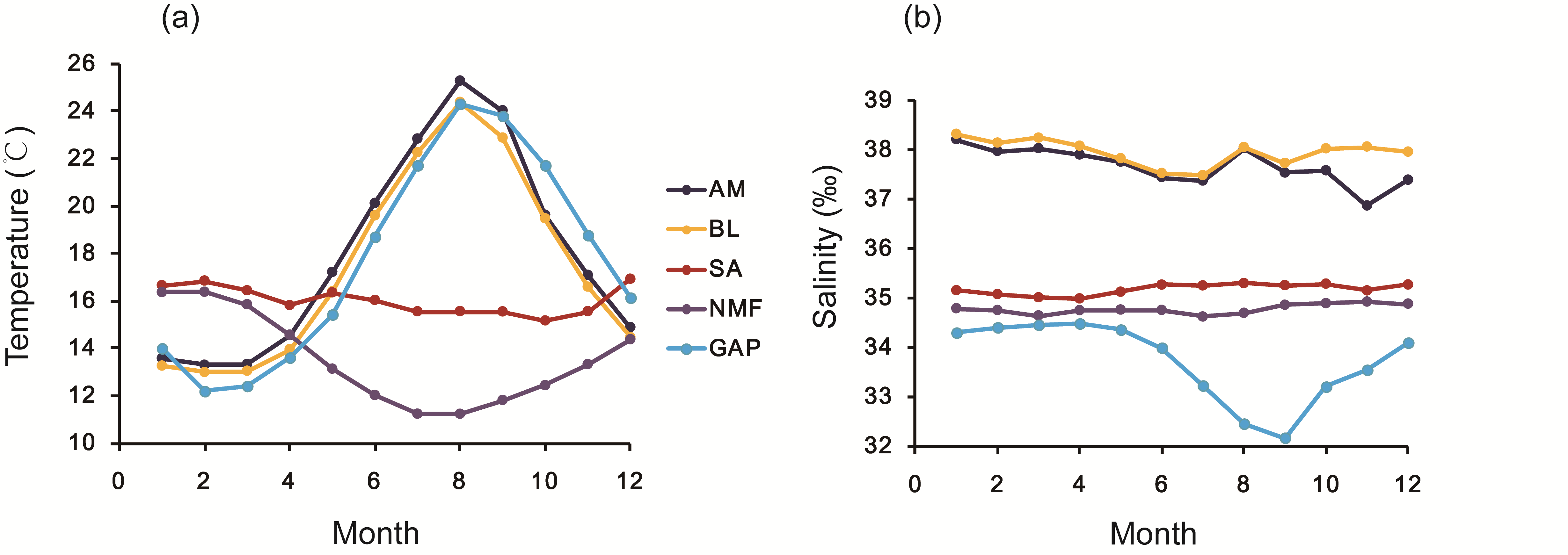


**Appendix S2** Environmental variables about sampling sites. Population names as per Table 1.


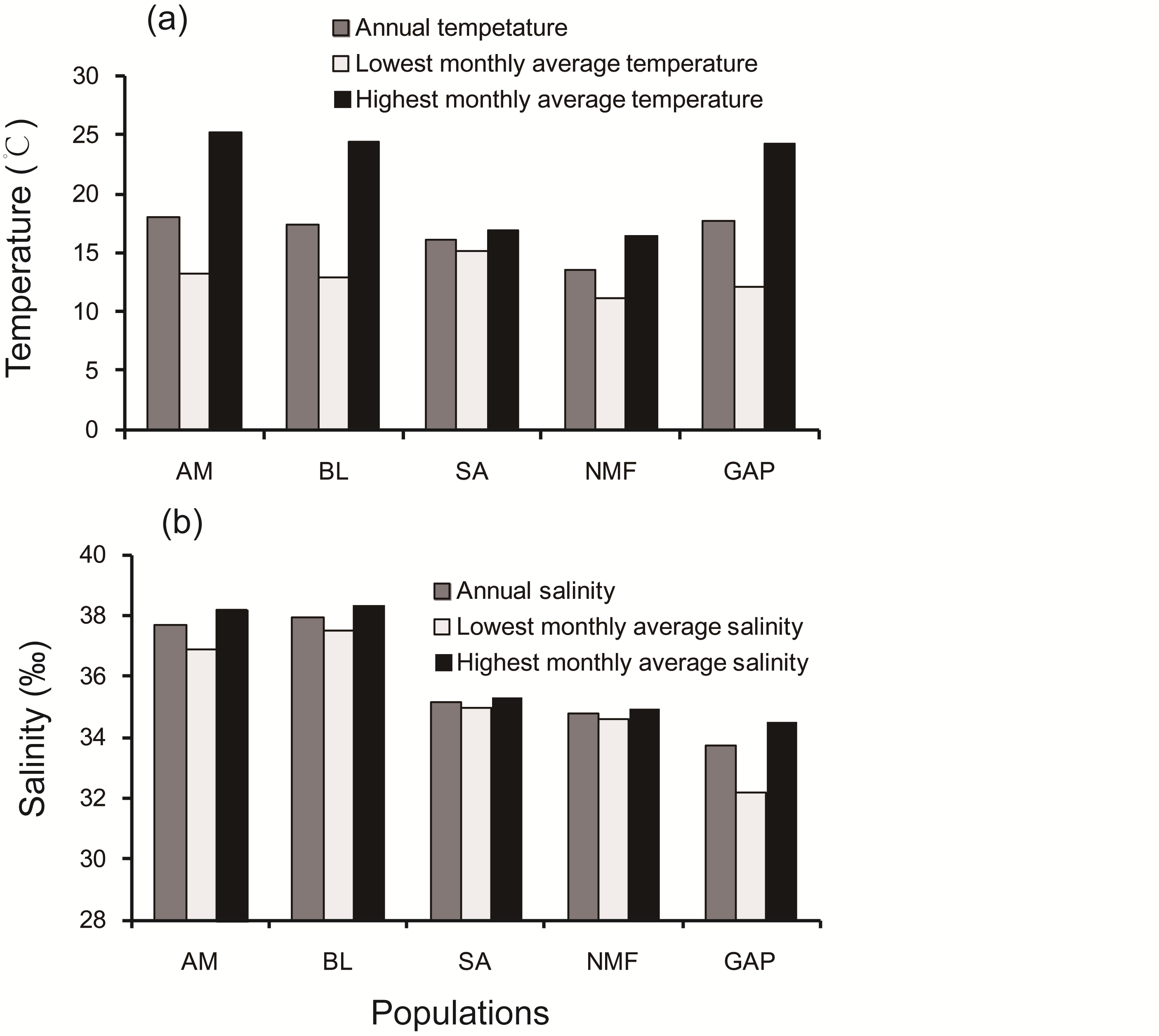


**Appendix S3** *P*-values for the exact test of the difference for the salinity (above diagonal) and temperature (below diagonal) using a nonparametric test (Mann-Whitney *U* test). **P* < 0.05; ***P* < 0.01; –, not significant.

|  | AM | BL | SA | NMF | GAP |
| --- | --- | --- | --- | --- | --- |
| AM |  | * | ** | ** | ** |
| BL | – |  | ** | ** | ** |
| SA | – | – |  | ** | ** |
| NMF | * | – | ** |  | ** |
| GAP | – | – | – | ** |  |

**Appendix S4** PCA plot on environmental variables for the studied *Ciona robusta* populations. Population names as per Table 1.


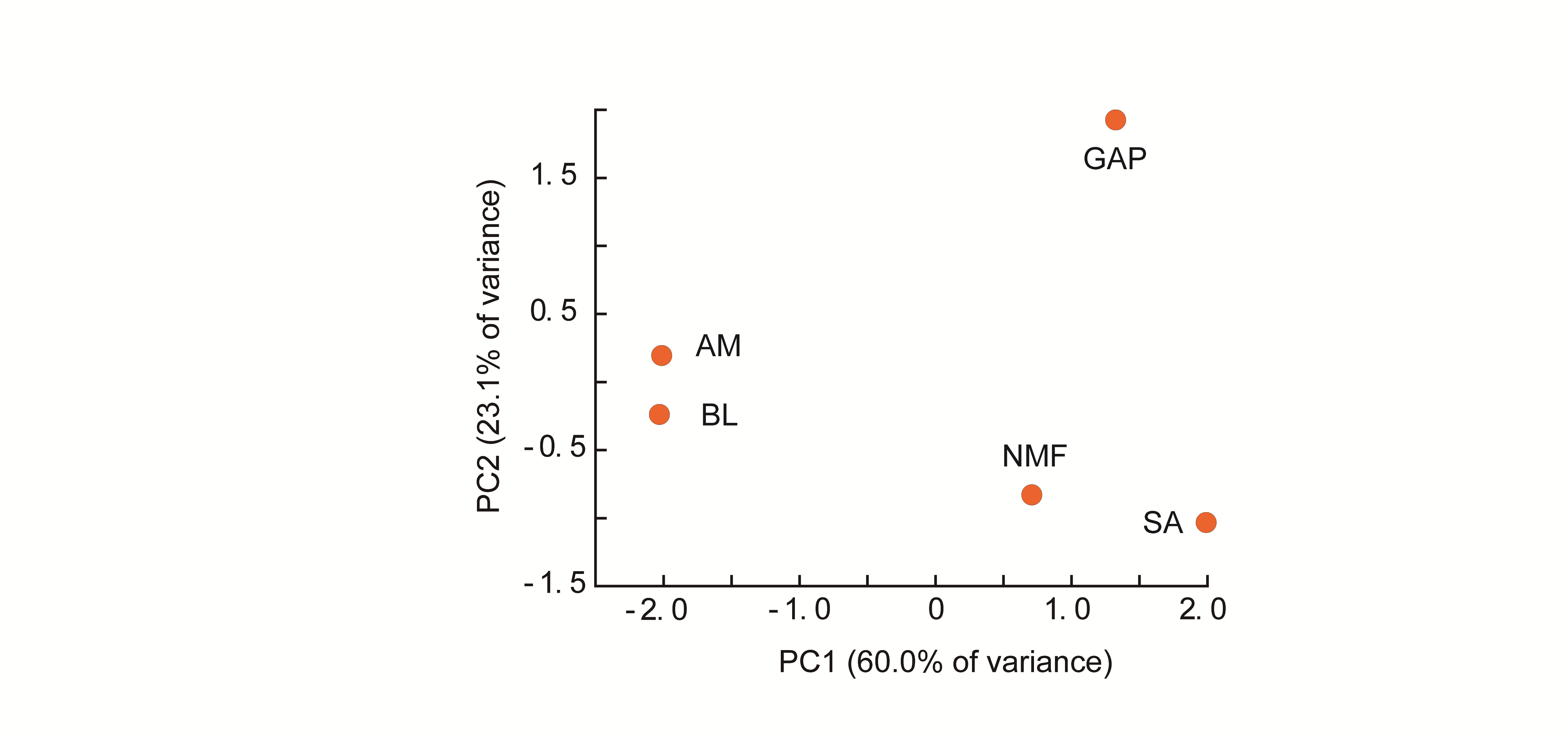


**Appendix S5** The results of Gene Ontology (GO) classification. The x-axis shows different categories of GO terms, while the y-axis shows the number of genes annotated into the corresponding term (right), and its proportion of the total number of annotated genes (left).


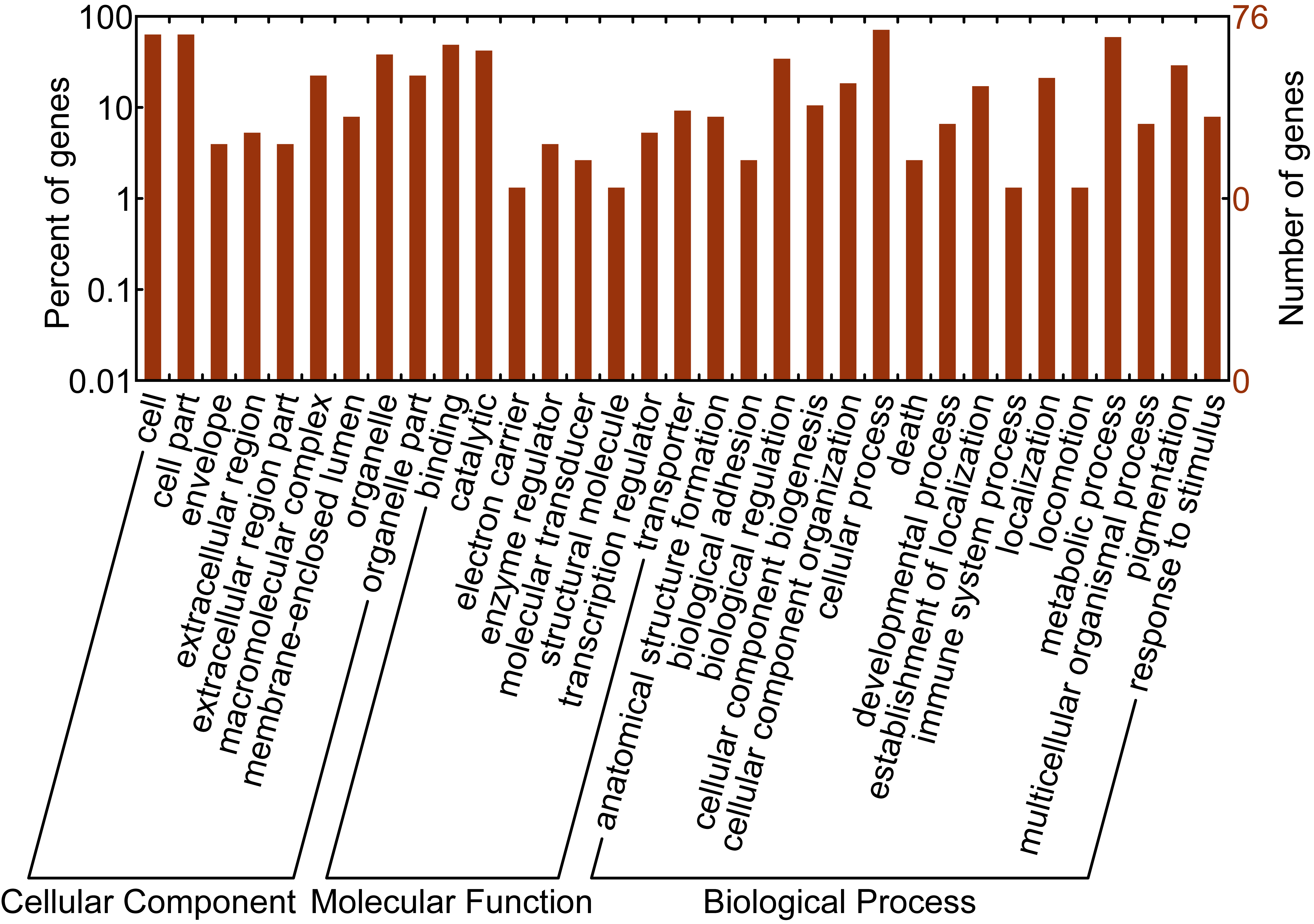


**Appendix S6** Basic information of microsatellite markers used for genome scan. CDS, coding sequence; *A*, number of observed alleles; *A*_R_, allelic richness; *H*_E_, expected heterozygosity; *F*_IS_, inbreeding coefficient. ^*^Significant deviation from Hardy-Weinberg equilibrium after Q values adjustment (*P* < 0.001).

| Locus | Position | *A* | *A*_R_ | *H*_O_ | *H*_E_ | *F*_IS_ |
| --- | --- | --- | --- | --- | --- | --- |
| Cin1 | CDS | 5 | 3.214 | 0.203 | 0.243 | 0.165 |
| Cin2 | 3'_UTR | 7 | 4.899 | 0.340 | 0.500 | 0.320^*^ |
| Cin3 | Unknown | 8 | 6.231 | 0.683 | 0.757 | 0.098 |
| Cin5 | CDS | 11 | 7.733 | 0.636 | 0.828 | 0.232^*^ |
| Cin6 | CDS | 9 | 5.485 | 0.704 | 0.702 | -0.003 |
| Cin8 | CDS | 10 | 5.555 | 0.406 | 0.682 | 0.406^*^ |
| Cin9 | 3'_UTR | 5 | 3.346 | 0.098 | 0.556 | 0.824^*^ |
| Cin10 | Unknown | 11 | 4.579 | 0.216 | 0.405 | 0.467 |
| Cin11 | CDS | 8 | 3.489 | 0.136 | 0.202 | 0.328 |
| Cin12 | 3'_UTR | 5 | 1.845 | 0.052 | 0.065 | 0.208 |
| Cin13 | CDS | 4 | 2.761 | 0.248 | 0.465 | 0.467^*^ |
| Cin14 | CDS | 5 | 1.890 | 0.042 | 0.069 | 0.389 |
| Cin15 | 3'_UTR | 11 | 6.406 | 0.607 | 0.726 | 0.164 |
| Cin17 | 3'_UTR | 4 | 2.379 | 0.094 | 0.191 | 0.511^*^ |
| Cin18 | CDS | 7 | 3.874 | 0.465 | 0.577 | 0.196 |
| Cin19 | CDS | 2 | 2.000 | 0.184 | 0.388 | 0.526 |
| Cin20 | 3'_UTR | 6 | 5.305 | 0.183 | 0.582 | 0.687^*^ |
| Cin21 | CDS | 12 | 7.160 | 0.662 | 0.748 | 0.116 |
| Cin22 | CDS | 5 | 3.657 | 0.798 | 0.632 | -0.266 |
| Cin24 | 3'_UTR | 7 | 4.012 | 0.224 | 0.396 | 0.435^*^ |
| Cin25 | CDS | 8 | 5.065 | 0.473 | 0.709 | 0.334^*^ |
| Cin27 | CDS | 4 | 3.750 | 0.546 | 0.660 | 0.172 |
| Cin32 | 3'_UTR | 7 | 3.326 | 0.102 | 0.429 | 0.764^*^ |
| Cin33 | Unknown | 10 | 6.181 | 0.244 | 0.729 | 0.666^*^ |
| Cin34 | Unknown | 5 | 3.245 | 0.458 | 0.550 | 0.168 |
| Cin35 | CDS | 20 | 8.888 | 0.370 | 0.738 | 0.499^*^ |
| Cin36 | CDS | 5 | 3.379 | 0.338 | 0.575 | 0.413 |
| Cin37 | CDS | 6 | 4.458 | 0.486 | 0.548 | 0.114 |
| Cin38 | CDS | 2 | 1.337 | 0.000 | 0.027 | 1.000^*^ |
| Cin39 | CDS | 4 | 1.604 | 0.021 | 0.049 | 0.565^*^ |
| Cin43 | CDS | 5 | 3.584 | 0.136 | 0.556 | 0.756^*^ |
| Cin48 | CDS | 3 | 2.546 | 0.243 | 0.220 | -0.106 |
| Cin54 | 3'_UTR | 6 | 4.179 | 0.189 | 0.467 | 0.597 |
| Cin56 | CDS | 4 | 2.553 | 0.311 | 0.502 | 0.381 |
| Cin58 | 3'_UTR | 9 | 6.114 | 0.395 | 0.763 | 0.483^*^ |
| Cin59 | CDS | 3 | 2.385 | 0.132 | 0.191 | 0.309 |
| Cin60 | 3'_UTR | 12 | 5.948 | 0.195 | 0.530 | 0.633^*^ |
| Cin61 | CDS | 3 | 2.912 | 0.525 | 0.572 | 0.081 |
| Cin66 | Unknown | 3 | 2.178 | 0.190 | 0.260 | 0.269 |
| Cin67 | Unknown | 5 | 4.320 | 0.162 | 0.710 | 0.772^*^ |
| Cin69 | Unknown | 8 | 2.860 | 0.022 | 0.148 | 0.851^*^ |
| Cin72 | 3'_UTR | 4 | 2.445 | 0.407 | 0.519 | 0.216 |
| Cin73 | CDS | 5 | 4.242 | 0.285 | 0.630 | 0.549^*^ |
| Cin74 | CDS | 6 | 4.783 | 0.137 | 0.594 | 0.769^*^ |
| Cin76 | CDS | 6 | 3.859 | 0.185 | 0.580 | 0.681^*^ |
| Cin77 | 3'_UTR | 8 | 3.191 | 0.329 | 0.447 | 0.265^*^ |
| Cin78 | 5'_UTR | 6 | 4.345 | 0.245 | 0.410 | 0.404 |
| Cin80 | CDS | 6 | 2.313 | 0.103 | 0.118 | 0.132 |
| Cin81 | 3'_UTR | 5 | 3.000 | 0.215 | 0.250 | 0.140 |
| Cin82 | CDS | 3 | 2.188 | 0.507 | 0.502 | -0.011 |
| Cin84 | 3'_UTR | 12 | 4.940 | 0.361 | 0.581 | 0.380^*^ |
| Cin86 | CDS | 3 | 2.092 | 0.236 | 0.262 | 0.101 |
| Cin88 | 3'_UTR | 6 | 2.600 | 0.165 | 0.163 | -0.017 |
| Cin90 | CDS | 5 | 3.982 | 0.576 | 0.643 | 0.105 |
| Cin92 | 3'_UTR | 7 | 3.913 | 0.163 | 0.451 | 0.640^*^ |
| Cin93 | CDS | 13 | 6.059 | 0.421 | 0.581 | 0.276 |
| Cin94 | CDS | 5 | 3.237 | 0.447 | 0.590 | 0.243^*^ |
| Cin95 | 3'_UTR | 4 | 2.397 | 0.031 | 0.346 | 0.911 |
| Cin96 | CDS | 5 | 3.217 | 0.451 | 0.495 | 0.090 |
| Cin97 | CDS | 15 | 10.035 | 0.738 | 0.872 | 0.155 |
| Cin98 | CDS | 5 | 3.078 | 0.162 | 0.221 | 0.266 |
| Cin101 | 3'_UTR | 6 | 4.210 | 0.357 | 0.448 | 0.204 |
| Cin102 | CDS | 3 | 2.414 | 0.240 | 0.240 | -0.002 |
| Cin103 | CDS | 4 | 2.902 | 0.160 | 0.206 | 0.224 |
| Cin104 | CDS | 4 | 1.872 | 0.047 | 0.072 | 0.350 |
| Cin105 | CDS | 8 | 4.379 | 0.490 | 0.580 | 0.156 |
| Cin106 | CDS | 4 | 2.448 | 0.161 | 0.300 | 0.465 |
| Cin107 | Unknown | 5 | 2.700 | 0.092 | 0.161 | 0.428 |
| Cin109 | CDS | 6 | 4.039 | 0.650 | 0.656 | 0.009 |
| Cin111 | 3'_UTR | 8 | 6.106 | 0.410 | 0.745 | 0.450^*^ |
| Cin112 | CDS | 6 | 4.142 | 0.324 | 0.498 | 0.351 |
| Cin115 | CDS | 3 | 2.199 | 0.271 | 0.370 | 0.270^*^ |
| Cin116 | Unknown | 4 | 3.404 | 0.212 | 0.604 | 0.650^*^ |
| Cin117 | CDS | 11 | 7.517 | 0.789 | 0.827 | 0.046 |
| Cin118 | CDS | 5 | 4.268 | 0.464 | 0.660 | 0.298^*^ |
| Cin119 | CDS | 5 | 4.255 | 0.553 | 0.669 | 0.174 |
| Cin122 | CDS | 4 | 2.207 | 0.444 | 0.440 | -0.009 |
| Cin123 | CDS | 4 | 2.225 | 0.162 | 0.175 | 0.078 |
| Cin124 | CDS | 9 | 5.311 | 0.200 | 0.605 | 0.670^*^ |
| Cin125 | 3'_UTR | 4 | 3.462 | 0.375 | 0.571 | 0.344^*^ |
| Cin126 | 3'_UTR | 5 | 3.016 | 0.437 | 0.550 | 0.207 |
| Cin127 | 3'_UTR | 5 | 2.243 | 0.077 | 0.126 | 0.392^*^ |
| Cin128 | CDS | 7 | 3.827 | 0.549 | 0.586 | 0.063 |
| Cin130 | CDS | 11 | 7.388 | 0.449 | 0.825 | 0.457^*^ |
| Cin132 | 5'_UTR | 6 | 3.936 | 0.119 | 0.584 | 0.798^*^ |
| Cin137 | CDS | 6 | 3.685 | 0.277 | 0.520 | 0.469 |
| Cin138 | CDS | 3 | 2.790 | 0.181 | 0.373 | 0.517 |
| Cin139 | 3'_UTR | 4 | 3.247 | 0.128 | 0.530 | 0.760^*^ |
| Cin140 | Unknown | 8 | 4.467 | 0.287 | 0.366 | 0.217 |
| Cin141 | Unknown | 3 | 2.229 | 0.139 | 0.481 | 0.712^*^ |
| Cin143 | 3'_UTR | 3 | 2.194 | 0.029 | 0.378 | 0.923^*^ |
| Cin144 | CDS | 9 | 5.087 | 0.466 | 0.502 | 0.072 |
| Cin146 | CDS | 5 | 3.441 | 0.303 | 0.398 | 0.239^*^ |
| Cin148 | 3'_UTR | 3 | 2.026 | 0.107 | 0.109 | 0.018 |
| Cin149 | 3'_UTR | 5 | 2.585 | 0.129 | 0.149 | 0.131 |
| Cin152 | 3'_UTR | 3 | 2.341 | 0.282 | 0.440 | 0.361^*^ |
| Cin153 | CDS | 3 | 2.523 | 0.333 | 0.514 | 0.352 |
| Cin155 | 5'_UTR | 3 | 2.729 | 0.351 | 0.355 | 0.011 |
| Cin157 | CDS | 6 | 4.494 | 0.237 | 0.566 | 0.582^*^ |
| Cin158 | CDS | 8 | 5.157 | 0.646 | 0.703 | 0.081 |
| Cin159 | 5'_UTR | 5 | 2.393 | 0.324 | 0.470 | 0.312 |
| Cin160 | CDS | 11 | 6.323 | 0.503 | 0.625 | 0.195 |
| Cin161 | CDS | 18 | 9.526 | 0.688 | 0.841 | 0.183 |
| Cin162 | 3'_UTR | 9 | 5.470 | 0.084 | 0.720 | 0.884^*^ |
| Cin163 | 3'_UTR | 3 | 2.062 | 0.138 | 0.142 | 0.026 |
| Cin164 | CDS | 6 | 4.076 | 0.275 | 0.497 | 0.446^*^ |
| Cin165 | CDS | 16 | 7.949 | 0.561 | 0.688 | 0.185 |
| Cin168 | CDS | 6 | 2.662 | 0.093 | 0.226 | 0.589^*^ |
| Cin170 | 5'_UTR | 10 | 7.088 | 0.215 | 0.808 | 0.734^*^ |
| Cin171 | 3'_UTR | 4 | 3.451 | 0.062 | 0.584 | 0.895^*^ |
| Cin172 | CDS | 8 | 5.797 | 0.654 | 0.712 | 0.082 |
| Cin173 | 3'_UTR | 9 | 5.280 | 0.417 | 0.710 | 0.413^*^ |
| Cin174 | CDS | 2 | 2.000 | 0.619 | 0.484 | -0.282 |
| Cin175 | CDS | 6 | 3.073 | 0.204 | 0.297 | 0.313 |
| Cin176 | CDS | 10 | 5.305 | 0.611 | 0.624 | 0.020 |
| Cin177 | CDS | 7 | 5.601 | 0.539 | 0.770 | 0.301^*^ |
| Cin178 | CDS | 3 | 1.386 | 0.014 | 0.029 | 0.497 |
| Cin179 | 3'_UTR | 5 | 3.285 | 0.514 | 0.617 | 0.166 |
| Cin181 | CDS | 13 | 6.153 | 0.457 | 0.721 | 0.367^*^ |
| Cin182 | CDS | 3 | 2.981 | 0.071 | 0.599 | 0.882^*^ |
| Cin183 | 3'_UTR | 8 | 4.514 | 0.214 | 0.620 | 0.655^*^ |
| Cin187 | CDS | 4 | 3.067 | 0.154 | 0.220 | 0.300 |
| Cin188 | 5'_UTR | 8 | 4.432 | 0.471 | 0.678 | 0.306^*^ |
| Cin189 | CDS | 8 | 4.817 | 0.172 | 0.707 | 0.757^*^ |
| Cin190 | 3'_UTR | 10 | 5.945 | 0.455 | 0.760 | 0.402^*^ |
| Cin192 | CDS | 8 | 5.022 | 0.155 | 0.545 | 0.717^*^ |
| Cin193 | CDS | 7 | 5.131 | 0.690 | 0.715 | 0.035 |
| Cin194 | Unknown | 7 | 3.307 | 0.258 | 0.552 | 0.534^*^ |
| Cin197 | CDS | 7 | 4.729 | 0.437 | 0.504 | 0.134 |
| Cin198 | CDS | 6 | 3.532 | 0.194 | 0.276 | 0.296 |
| Cin200 | CDS | 4 | 3.050 | 0.110 | 0.524 | 0.790^*^ |
| Cin201 | CDS | 4 | 2.292 | 0.119 | 0.272 | 0.565 |
| Cin202 | CDS | 5 | 3.367 | 0.500 | 0.649 | 0.231 |
| Cin203 | Unknown | 7 | 3.631 | 0.434 | 0.453 | 0.041 |
| Cin204 | 3'_UTR | 16 | 8.967 | 0.723 | 0.816 | 0.114^*^ |
| Cin205 | Unknown | 5 | 2.910 | 0.313 | 0.517 | 0.395^*^ |
| Cin207 | CDS | 7 | 4.980 | 0.242 | 0.593 | 0.592^*^ |
| Cin208 | 3'_UTR | 11 | 4.874 | 0.224 | 0.311 | 0.281 |
| Cin210 | Unknown | 10 | 4.773 | 0.156 | 0.676 | 0.770^*^ |
| Cin211 | Unknown | 7 | 4.566 | 0.357 | 0.629 | 0.433^*^ |
| Cin212 | Unknown | 5 | 2.902 | 0.132 | 0.222 | 0.407^*^ |
| Cin213 | Unknown | 12 | 8.027 | 0.223 | 0.846 | 0.737^*^ |
| Cin215 | CDS | 8 | 5.129 | 0.388 | 0.610 | 0.364^*^ |
| Cin216 | Unknown | 4 | 2.557 | 0.072 | 0.145 | 0.500^*^ |
| Cin219 | CDS | 15 | 7.254 | 0.388 | 0.790 | 0.510^*^ |
| Cin221 | CDS | 4 | 2.491 | 0.172 | 0.162 | -0.065 |
| Cin222 | CDS | 8 | 5.379 | 0.471 | 0.754 | 0.376^*^ |
| Cin225 | 3'_UTR | 4 | 3.068 | 0.077 | 0.441 | 0.826^*^ |
| Cin226 | CDS | 9 | 6.092 | 0.657 | 0.670 | 0.019 |
| Cin227 | 3'_UTR | 12 | 4.793 | 0.548 | 0.611 | 0.104 |
| Cin228 | Unknown | 8 | 4.951 | 0.132 | 0.503 | 0.737^*^ |
| Cin229 | CDS | 3 | 2.872 | 0.250 | 0.325 | 0.232 |

**Appendix S7** Individual Bayesian assignment proportions based on STRUCTURE for (a) all tested loci, (b) neutral loci, (c) loci under balancing selection, and (d) directionally selected loci in all populations. *K*-values were determined based on the *ΔK* method of Evanno *et al*.^57^.


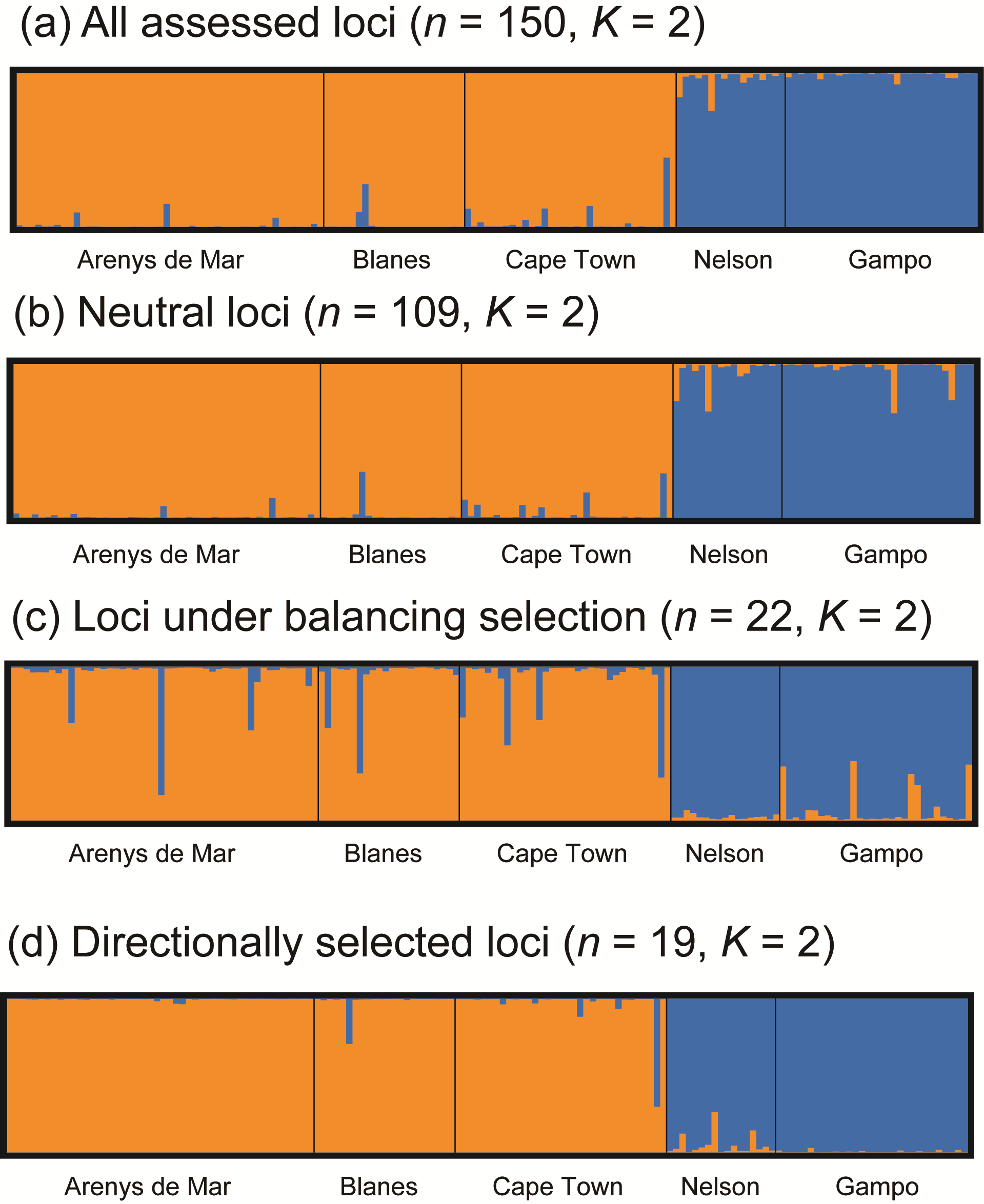


**Appendix S8** Loci under directional selection based on BAYESCAN for population pairwise analysis (95% confidence). The loci detected only in population pairwise analysis are bolded.

| Locus | Annotation | Populations |
| --- | --- | --- |
| **Cin8** | **LOC100180162** | **NMF, GAP** |
| Cin19 | – | SA, NMF |
| Cin20 | – | AM, SA, NMF |
| Cin27 | – | AM, BL, SA, NMF, GAP |
| Cin54 | – | AM, BL, SA, NMF, GAP |
| Cin74 | – | BL, SA, NMF |
| Cin95 | – | AM, BL, SA, NMF, GAP |
| Cin106 | – | AM, NMF |
| **Cin124** | **E3 SUMO-protein ligase RanBP2-like** | **NMF, GAP** |
| Cin138 | – | AM, SA, GAP |
| Cin189 | – | AM, BL, SA, NMF, GAP |

**Appendix S9** Outlier detection at 150 loci with BAYESCAN based on population pairwise analysis.


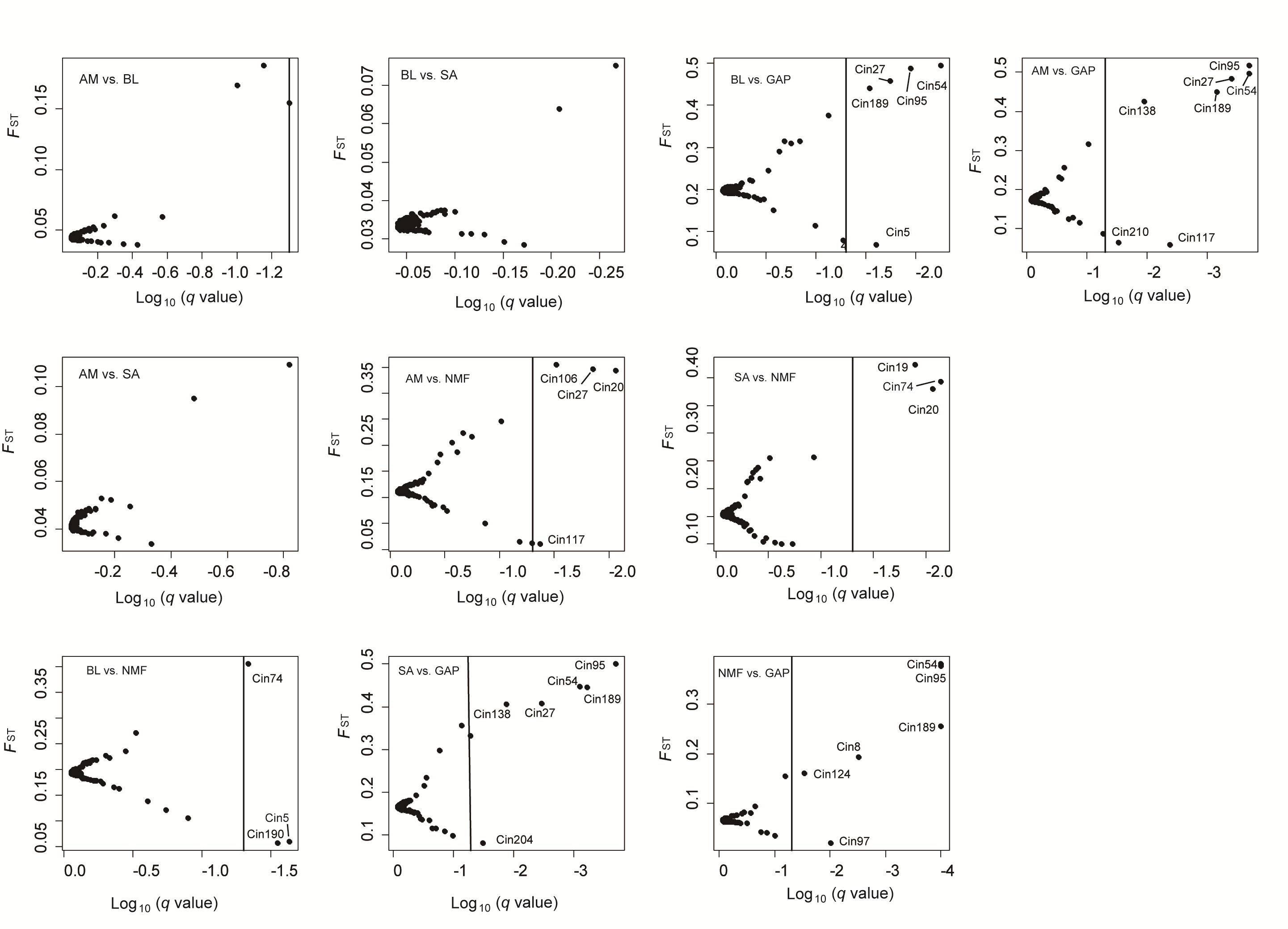


**Appendix S10** Summary of loci under balancing selection in LOSITAN, BAYESCAN and ARLEQUIN analyses based on the global analysis. IAM, infinite alleles model; SMM, stepwise-mutation model; **P* < 0.05; ***P* < 0.01; –, not significant. The loci detected consistently across all the three methods (i.e. LOSITAN, BAYESCAN and ARLEQUIN) are bolded.

|  |  | LOSITAN | |  |  |
| --- | --- | --- | --- | --- | --- |
| Locus | Annotation | IAM | SMM | ARLEQUIN | BAYESCAN |
| Cin5 | SCO-spondin | – | – | – | ** |
| **Cin6** | **immunoglobulin superfamily member 10-like** | ***** | ***** | ***** | ****** |
| Cin13 | protein GREB1-like | * | * | * | – |
| **Cin35** | **mediator of RNA polymerase II transcription subunit 15** | ***** | ***** | ***** | ***** |
| Cin84 | protein YIPF4-like | – | * | – | – |
| Cin94 | class I cytokine receptor glycoprotein 130-like | ** | ** | ** | – |
| Cin109 | cyclin-T1 | – | * | * | – |
| **Cin117** | **procollagen C-endopeptidase enhancer 1-like** | ****** | ****** | ****** | ****** |
| Cin128 | zinc finger protein 420 | * | * | * | – |
| Cin141 | no hit | * | * | * | – |
| **Cin146** | **leucine-rich repeat transmembrane neuronal protein 2** | ****** | ***** | ***** | ***** |
| Cin148 | regulator of G-protein signaling 21-like | ** | ** | * | – |
| Cin158 | uncharacterized protein LOC100180738 | ** | ** | * | – |
| Cin168 | uncharacterized protein LOC100185324 | * | ** | * | – |
| Cin178 | poly(A)-specific ribonuclease PARN | ** | * | – | – |
| Cin179 | mitochondrial carnitine/acylcarnitine carrier protein-like | – | * | – | – |
| Cin190 | serine/threonine-protein kinase N2-like | – | – | – | * |
| Cin193 | CREB-binding protein | * | * | * | – |
| **Cin210** | **no hit** | ***** | ***** | ***** | ****** |
| Cin219 | uncharacterized LOC101242065 | * | * | * | – |

**Appendix S11** Loci under balancing selection based on BAYESCAN for population pairwise comparisons (95% confidence). The loci detected only in population pairwise analysis are bolded.

| Locus | Annotation | Populations |
| --- | --- | --- |
| Cin5 | – | BL, NMF, GAP |
| **Cin97** | **cell wall integrity and stress response component 2** | **NMF, GAP** |
| Cin117 | – | AM, NMF, GAP |
| Cin190 | – | BL, NMF |
| **Cin204** | **cGMP-inhibited 3',5'-cyclic phosphodiesterase A** | **SA, GAP** |
| Cin210 | – | AM, GAP |

**Appendix** **S12** Schematic representation of the genes in selective sweep windows within a 20-kb distance up- and down-stream at 19 loci under directional selection (red). The location for each gene was assessed by BLAST against the KH assembly by Satou *et al*.^18^.


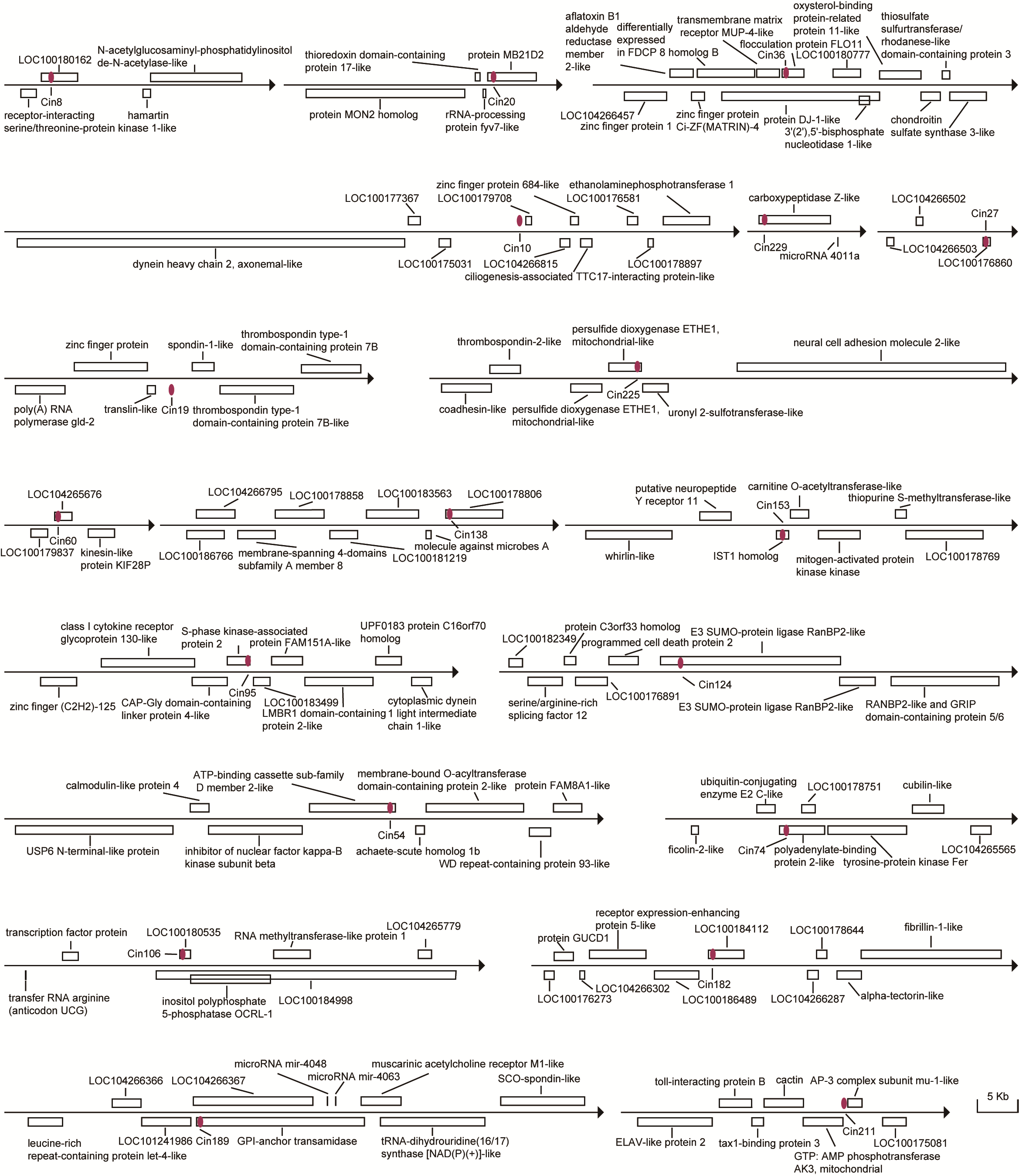


**Appendix S13**. Detailed results for SAM analysis for microsatellite loci with at least one allele that is significantly associated with one of the environmental variables tested (*P*<0.001, Bonferroni correction for multiple comparisons). The putatively selected loci are indicated in bold text.

| Locus | Annual temperature | Minimum temperature | Maximum temperature | Annual salinity | Minimum salinity | Maximum salinity |
| --- | --- | --- | --- | --- | --- | --- |
| Cin3 | – | – | – | 199 | 199, 223 | – |
| **Cin8** | 346 | – | 346, 358 | – | – | – |
| **Cin10** | – | – | – | – | 362 | – |
| Cin18 | – | 175 | – | – | 175, 178 | – |
| **Cin19** | – | 240 | – | – | 240 | – |
| **Cin20** | 229 | 233 | – | – | 233 | – |
| **Cin27** | – | 132, 141 | – | 135 | 135 | 135 |
| Cin33 | – | – | – | – | 215 | – |
| **Cin36** | – | 175 | – | 175, 205 | 175, 205 | 205 |
| **Cin60** | – | – | – | – | 188 | – |
| Cin66 | – | 298 | – | – | 298 | – |
| Cin73 | – | – | – | – | 197 | – |
| **Cin74** | – | 238 | – | 238 | 238 | – |
| Cin76 | – | 544 | – | – | – | – |
| Cin93 | – | 250 | – | – | – | – |
| Cin105 | – | – | – | – | 358 | – |
| **Cin106** | – | 281 | – | – | 281 | – |
| Cin112 | – | 386 | – | – | – | – |
| Cin137 | – | – | – | – | 296, 298 | – |
| **Cin138** | – | 353 | – | 353 | 350, 353 | 353 |
| **Cin153** | – | – | – | 267, 270 | 267, 270 | 270 |
| Cin161 | – | 195 | – | – | 189, 192 | – |
| Cin162 | – | – | – | 205 | 173 | – |
| Cin177 | – | 245 | – | – | 245 | – |
| Cin181 | – | 259 | 259 | 217 | 217 | 217 |
| Cin183 | – | – | – | – | 258 | – |
| **Cin189** | – | – | – | – | 232 | – |
| Cin197 | – | – | 231 | – | – | – |
| **Cin204** | – | – | – | – | – | 290 |
| **Cin211** | – | 168 | – | 168 | 168 | 168 |
| Cin215 | – | 247 | – | – | 247 | – |
| **Cin225** | – | – | – | – | 212 | – |
| **Cin229** | – | 309 | – | – | 309 | – |
